# Regulatory Mechanisms Underlying the Expression of Prolactin Receptor in Chicken Granulosa Cells

**DOI:** 10.1371/journal.pone.0170409

**Published:** 2017-01-20

**Authors:** Shenqiang Hu, Raj Duggavathi, David Zadworny

**Affiliations:** Department of Animal Science, McGill University, Macdonald Campus, Ste. Anne de Bellevue, Quebec, Canada; Baylor College of Medicine, UNITED STATES

## Abstract

Prolactin (PRL) has both pro- and anti-gonadal roles in the regulation of avian ovarian functions through its interaction with the receptor (PRLR). However, neither the pattern of expression of PRLR nor its regulatory mechanisms during follicle development have been clearly defined. The objective of the present study was to investigate mechanisms of *PRLR* expression in chicken granulosa cells. Levels of *PRLR* transcript were highest in the stroma and walls of follicles < 2 mm in diameter and progressively declined with the maturation of follicles. In preovulatory follicles, *PRLR* was expressed at higher levels in granulosa than theca layers. FSH exerted the greatest stimulatory effect on *PRLR* and *StAR* expression in cultured granulosa cells of the 6–8 mm follicles but this effect declined as follicles matured to F1. In contrast, LH did not alter the expression of *PRLR* in granulosa cells of all follicular classes but increased levels of *StAR* in F2 and F1 granulosa cells. Both non-glycosylated- (NG-) and glycosylated- (G-) PRL upregulated basal *PRLR* expression in granulosa cells of the 6–8 mm, F3 or F1 follicles but had little effect in F2 follicles. Furthermore, FSH-stimulated *PRLR* expression was reduced by the addition of either isoform of PRL especially in F2 granulosa cells. These results indicate that *PRLR* is differentially distributed and regulated by FSH or PRL variants independently or in combination in the follicular hierarchy. By using activators and inhibitors, we further demonstrated that multiple signaling pathways, including PKA, PKC, PI3K, mTOR and AMPK, are not only directly involved in, but they can also converge to modulate ERK2 activity to regulate FSH-mediated *PRLR* and *StAR* expression in undifferentiated granulosa cells. These data provide new insights into the regulatory mechanisms controlling the expression of *PRLR* in granulosa cells.

## Introduction

In chickens, ovarian follicles go through initial (activation of cortical follicles) and cyclic (follicle selection) recruitment before ovulation. These events are tightly coupled with the morphological and functional changes in granulosa cells [1]. In follicles prior to selection, granulosa cells are undifferentiated and steroidogenically inactive [2] due to low levels of expression of the two key genes required for steroidogenesis, steroidogenic acute regulatory protein (StAR) [3] and cytochrome P450 side chain cleavage (P450_scc_) enzyme [4]. Subsequent to selection, granulosa cells are differentiated and become steroidogenically active [5]. The process of follicle selection is mainly under the control of follicle stimulating hormone (FSH) [5, 6]. Within the cohort of prehierarchical 6–8 mm follicles, a single follicle showing the highest expression of FSH receptor (FSHR) in the granulosa layer is likely to be next in line to enter the preovulatory hierarchy [7]. FSH signaling leads to the differentiation of granulosa cells by controlling the expression of several steroidogenic genes such as *StAR*, *P450*_*scc*_ and luteinizing hormone receptor (*LHR*), which is achieved via modulation of multiple intracellular signaling cascades, including protein kinase A (PKA), protein kinase C (PKC), phosphatidylinositol 3-kinase (PI3K) and extracellular signal-regulated kinases (ERKs) and AMP-activated protein kinase (AMPK) [1, 5, 8, 9]. In differentiated granulosa cells, LHR substitutes for the dominant role of FSHR in further promoting LH-induced steroidogenesis which is largely mediated by the PKA pathway [5].

Evidence is accumulating that in addition to gonadotropins, prolactin (PRL) may also play a critical role in the follicular hierarchy in birds. The anti-gonadal effects of PRL are substantiated by the strong association of high PRL secretion with degeneration and disappearance of the follicular hierarchy during incubation in many species of birds. In broody breeds of chickens and turkeys, intramuscular injections of 10–160 IU of PRL cause significant decreases in ovarian weight and the number of normal follicles but an increase in the number of atretic follicles [10]. These effects are indirect via inhibition of the hypothalamo-adenohypophyseal axis and the release of LH [11, 12] and direct since PRL suppresses both basal and gonadotropin-induced levels of progesterone and estradiol production by the ovary [13]. In contrast, neutralization or inhibition of endogenous PRL via passive or active immunizations against PRL or its releasing factor, vasoactive intestinal peptide (VIP), attenuated the expression of incubation behavior and improved egg production performance [14–18]. Nevertheless, the observations that plasma PRL levels gradually increase before the onset of sexual maturity and egg laying still occurs during early stages of incubation in birds also imply that PRL below a threshold concentration appears to be pro-gonadal. Indeed, in less- or non-broody chickens, immunizations against PRL or its receptor (PRLR) depressed egg production by reducing large white follicular growth and hence recruitment into the follicular hierarchy [19]. Furthermore, *in vitro* effects of PRL on steroid secretion by cultured ovarian follicles are stimulatory or inhibitory dependant on the concentration of PRL, the type of follicular cells and the stages of follicle development as well as the stage of the ovulatory process [20]. Nevertheless, so far little is known about the involvement of the PRL-PRLR system in the process of follicle selection as well as how it is regulated in birds.

It is well known that PRL exerts its effects through interaction with the receptor, PRLR [21]. Despite extremely low or even undetectable levels of *PRL* transcript in the chicken ovary [22–24], *PRLR* mRNA is abundant in the ovaries of chickens [25] and turkeys [26]. In particular, *PRLR* transcript is expressed at higher levels in walls of small follicles than those of large follicles in turkeys [26]. Therefore, it is likely that PRL may affect the follicular hierarchy mainly in an endocrine manner. However, the expression pattern of *PRLR* in cell type or follicular size classes during follicle development in chickens has not been investigated. In addition, post-translational modification contributes to different forms of circulating PRL in birds and glycosylated (G-) PRL is a major isoform dependent on the stage of the reproductive cycle. Since glycosylation is able to modulate the biological activity of PRL by influencing its receptor-binding efficiency [21] and the ratio of G- to non-glycosylated (NG-) PRL varies during various reproductive stages in chickens [27] and turkeys [28], interactions between G-, NG-PRL and PRLR may occur to partition the effects of PRL on ovarian follicles. Thus, it is of importance to investigate the effect of PRL glycosylation on PRLR expression during follicle development. The objectives of the present study were: 1) to determine the expression profile and cellular distribution of *PRLR* during chicken follicle development; 2) to investigate the effects of gonadotropins on *PRLR* expression; 3) to examine the role of NG- and G-PRL in basal and gonadotropin-regulated *PRLR* expression; 4) to elucidate the cellular mechanisms controlling the modulatory effects of FSH on *PRLR* expression by cultured chicken granulosa cells.

## Materials and Methods

### Hormones, chemicals and reagents

Recombinant human FSH (rhFSH, AFP8468A), ovine LH (oLH, AFP-5551B), non-glycosylated ovine PRL (NG-oPRL, AFP-10692C) and glycosylated ovine PRL (G-oPRL, AFP-5742B) were obtained from National Hormone & Peptide Program (Torrance, CA, USA). A stock solution of each hormone was correspondingly prepared with either PBS at appropriate pH or 0.01M NaHCO_3_, and then stored in small aliquots at -80℃. The final concentration of each working solution used for corresponding treatment was prepared with culture medium. Protein kinase A (PKA) activator (Forskolin) and inhibitor (H89), protein kinase C (PKC) activator (Phorbol 12-myristate 13-acetate, PMA) and inhibitor (GF109203X), phosphatidylinositol 3-kinase (PI3K) inhibitor (LY294002), mammalian target of rapamycin (mTOR) inhibitor (Rapamycin), extracellular signal-regulated kinase (ERK) kinase (MEK) inhibitor (PD0325901) and AMP-activated protein kinase (AMPK) activator (AICAR) were purchased from Selleck Chemicals (Houston, TX, USA). Fetal bovine serum (FBS), Dulbecco’s Modified Eagle’s Medium/Nutrient Mixture F12 (DMEM/F12), 0.4% Trypan Blue Solution as well as penicillin and streptomycin mixture were purchased from Invitrogen Life technologies (Carlsbad, CA, USA). Type Ⅱ collagenase as well as phosphatase and protease inhibitor cocktail were purchased from Sigma-Aldrich (Oakville, ON, Canada). Trizol reagent and high-capacity cDNA reverse transcription kit were purchased from Invitrogen Life technologies (Carlsbad, CA, USA). Power SYBR Green PCR Master Mix was purchased from D-Mark Bioscience (Toronto, ON, Canada). Protein Assay Kit, Bovine Serum Albumin (BSA), 4 × Laemmli buffer, 2-mercaptoethanol and Immun-Star Western C Chemiluminescent Kit were purchased from Bio-Rad Laboratories (Hercules, CA, USA). Rabbit monoclonal anti-phospho- and anti-total-ERK1/2 primary antibodies were purchased from Cell Signaling Technology (Beverly, MA, USA). A rabbit polyclonal anti-β-actin primary antibody and a horseradish peroxidase (HRP)-conjugated goat anti-rabbit IgG antibody were purchased from Abcam (Cambridge, UK).

### Animals and tissue collection

All experimental procedures using chickens in this study were approved by the Faculty Animal Care Committee of McGill University. White Leghorn hens, 25–35 weeks of age and laying actively, were used in all studies described. Hens were fed *ad libitum* and kept in individual cages under standard conditions at the Poultry Complex of Macdonald Campus Farm, McGill University. The time of oviposition was monitored for each hen using surveillance camera (Lorex corporation, Maryland, USA), and ovulation was predicted to occur within 15–30 min after oviposition of the previous egg in the laying sequence. Four hens were randomly selected for ovarian tissue collection and were killed approximately 1–4 h before predicted time of a mid-sequence ovulation by cervical dislocation. After slaughter, the ovary from each hen was immediately removed and placed into ice-cold 0.9% NaCl solution. According to the diameter and the position in the follicular hierarchy, ovarian follicles were categorized into several groups, including the stroma, prehierarchical (< 2, 2–4, 4–6 and 6–8 mm) and preovulatory (9–12 mm and F5-F1, 13–40 mm; F5 < F4 < F3 < F2 < F1) follicles. Follicular walls consisting of a mixture of cells including theca and granulosa cells were collected from the < 2 mm to F4 follicles, and theca and granulosa cell layers were isolated from F3-F1 follicles, respectively, according to the method as previously described [29]. To further determine changes in the expression of proteins of interest between undifferentiated and differentiated follicles, another 4 hens were sacrificed to collect the stroma, follicular walls from the < 2 to 4–6 mm follicles as well as the theca and granulosa cell layers isolated from each category of the 6–8 mm, F3, F2 and F1 follicles, respectively. All samples were snap frozen in liquid nitrogen and then stored at -80℃ until analysis.

### Granulosa cell culture and treatments

Granulosa cell layers harvested from each category of prehierarchical (6–8 mm) follicles as well as the three largest preovulatory follicles (F3, F2 and F1) were digested with 0.1% Type Ⅱ collagenase, respectively. The number of each category of granulosa cells were counted using a hemocytometer and cell viability was assessed by trypan blue exclusion test. The cells were then seeded onto 12-well culture plates at a density of ~3 × 10^5^ cells/well in 1 ml DMEM/F12 medium containing 3% FBS and 1% penicillin-streptomycin mixture. Cells were cultured at 38.5℃ in a humidified atmosphere of 95% air and 5% CO_2_. After 24 h of incubation with a medium change at 6 h, non-attached cells were removed by aspiration and adherent cells were washed three times with serum-free medium (DMEM/F12 supplemented with 1% penicillin-streptomycin mixture). Serum-free medium was used in all further incubations. The cells were subsequently treated with 10 ng/ml rhFSH, or 10 ng/ml oLH, or different concentrations of NG- and G-oPRL (0, 1, 10, 100 or 1000 ng/ml), or in combination for another 24 h. To further explore the involvement of signaling pathways, the cells were cultured in the absence or presence of 10 ng/ml rhFSH, or together with corresponding activator or inhibitor of intracellular signaling cascades for another 4 h. Finally, the culture media were removed and the cells were collected for either RNA isolation or protein extraction. Each *in vitro* experiment was independently performed at least three times using tissues from different hens.

### RNA isolation and real time PCR

Total RNA was extracted using Trizol reagent (Invitrogen, USA) according to the manufacturer’s protocol. The purity and concentration of RNA was determined using a NanoDrop Spectrophotometer (Thermo Fisher Scientific, Waltham, MA, USA), and the integrity of RNA was assessed by visualization of the 28S/18S rRNA ratio after electrophoresis on 1.5% agarose gels. The cDNA was then synthesized from 1 μg RNA using high-capacity cDNA reverse transcription kit (Invitrogen, USA). Real time quantitative PCR (qPCR) reactions were performed on the CFX384^TM^ real-time PCR detection system (Bio-Rad, USA) using SYBR Green master mix (D-Mark Bioscience, Ontario, Canada). Reactions were conducted with the following conditions: pre-denaturation at 95℃ for 5 min, followed by 40 cycles of denaturation at 95℃ for 15 s and annealing/extension at corresponding temperature of each primer set for 30 s. The no-template controls and negative controls without reverse transcriptase were also included in all qPCR runs. Target specificity for each primer set was validated by melting curve analyses. In addition, the identity of all amplicons was verified by sequencing. Standard curves were generated by 5-fold serial dilutions of cDNA to determine the amplification efficiency of PCR reactions. The efficiencies were nearly 100% and had a coefficient of determination (*R*^*2*^) > 0.98, allowing the use of the comparative Cq method (ΔΔCq) [30]. The *18S rRNA* gene was used as the internal control. All samples were amplified in triplicate and relative mRNA levels of target genes were normalized to *18S rRNA*. All qPCR results were shown as fold-differences in comparison to an appropriate reference tissue or untreated control. The primers used for real time qPCR are listed in Table 1.

**Table 1:** Primer pairs for real-time quantitative PCR.

Gene		Sequence (5’ to 3’)	Tm (℃)	GenBank Accession No.
*PRLR*	F	CCTTCCACCAGTGCTTCAA	56.4	NM_204854
	R	AGGAGGCTGACTGTTAGGT		
*StAR*	F	GTCCCTCGCAGACCAAGTT	59.8	NM_204686
	R	GGTGCTTGGCGAAGTCCA		
*18S rRNA*	F	TTAAGTCCCTGCCCTTTGTACAC	60	AF173612
	R	CGATCCGAGGAACCTCACTAAAC		

F, forward primer; R, reverse primer.

### Protein extraction and western blot analysis

Tissue and cell lysates were prepared in Triton X-100 lysis buffer containing 10 mM Tris-Hcl (pH 7.5), 5 mM EDTA (pH 8.0), 150 mM NaCl, 30 mM sodium pyrophosphate, 30 mM sodium fluoride, 1 mM sodium orthovanadate activated and 0.5% Triton X-100 as well as phosphatase and protease inhibitor cocktail. Protein concentration was determined by using Protein Assay Kit with Bovine Serum Albumin as the standard (Bio-Rad, USA). Equal amounts of protein lysates (20–40 μg) mixed with appropriate 4 × Laemmli buffer were boiled at 95℃ for 5 mins and then resolved by 10% sodium dodecyl sulfate (SDS)-polyacrylamide gel electrophoresis (PAGE). After being transferred to nitrocellulose membranes, the membranes were subsequently blocked with 5% skim milk and then incubated overnight at 4℃ with corresponding primary antibodies. Following being washed 3 times with TBST (10 mins each time), the membranes were further incubated with the secondary antibody for 1.5 h at room temperature and then washed 3 times with TBST. The immunoblotted proteins were finally detected with Immun-Star Western C Chemiluminescent Kit (Bio-Rad, USA) by using Chemidoc Analyzer (Bio-Rad, USA). With regard to proteins of similar size, the membranes were stripped using stripping buffer (20 ml 20% SDS; 12.5 ml 0.5 M Tris-Hcl, pH 6.8; 67.5 ml DEPC H_2_O and 0.8 ml 2-mercaptoethanol) and then washed with TBST, followed by being blocked with 5% skim milk before re-incubation with another primary antibody. The rabbit monoclonal anti-phospho- and anti-total-ERK1/2 primary antibodies were used at 1:1,000 dilution. The rabbit polyclonal anti-β-actin primary antibody was used at 1:5,000 dilution, while the horseradish peroxidase (HRP)-conjugated goat anti-rabbit IgG antibody was used at 1:10,000. Densitometric analysis was performed using Image Lab software (Version 4.1, Bio-Rad), and the relative abundance of phospho-ERK2 was normalized to total ERK2 and then was expressed as fold changes compared to an appropriate tissue or untreated control.

### Statistical analyses

All results are expressed as mean ± SEM of a minimum of three independent experiments. Statistical comparisons between groups were analyzed by analysis of variance (ANOVA) followed by Tukey’s test using SAS 9.4 (SAS Institute, Cary, USA). A p-value less than 0.05 was considered statistically significant.

## Results

### Expression pattern of *PRLR* mRNA in chicken ovarian follicles

**Fig 1:**
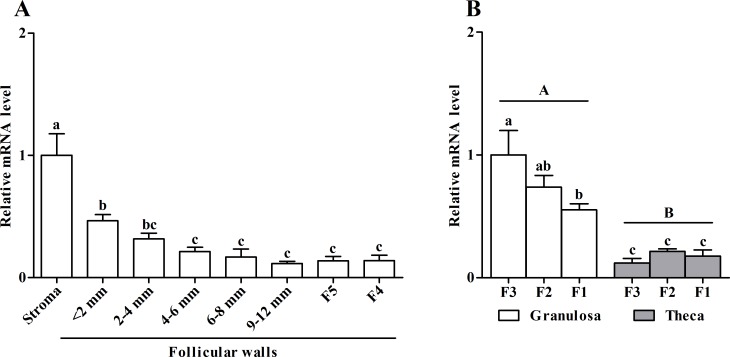
Expression pattern of *PRLR* mRNA in chicken developing follicles. (A) Abundance of *PRLR* mRNA in the stroma and walls of prehierarchical (< 2, 2–4, 4–6 and 6–8 mm) and preovulatory (9–12 mm, F5 and F4) follicles. (B) Relative *PRLR* mRNA levels in theca and granulosa cell layers isolated from the three largest preovulatory follicles (namely F3-F1; F3 < F2 < F1). Relative expression level was normalized to *18S rRNA*. Data are expressed as fold differences ± SEM compared to either the stroma or F3 granulosa cells (n = 4 hens). Different lowercase letters indicate a significant effect of follicular developmental stage, whilst different uppercase letters indicate a significant effect of cell type (granulosa versus theca cell layer). *P* < 0.05 was accepted as statistically significant.

Changes in levels of *PRLR* mRNA in the stroma, prehierarchical (< 2, 2–4, 4–6 and 6–8 mm) and preovulatory (9–12 mm and F5-F1; F5 < F4 < F3 < F2 < F1) follicles are presented in Fig 1. The highest levels of *PRLR* mRNA were observed in the stroma and follicles < 2 mm in diameter. Subsequently, their levels progressively declined as the follicles developed (Fig 1A). In preovulatory follicles (F3-F1), *PRLR* transcript was more abundant in granulosa than theca layers and their levels decreased in granulosa layers as the follicles approached to the largest diameter (Fig 1B).

### Gonadotropin regulation of *PRLR* expression in chicken granulosa cells

**Fig 2:**
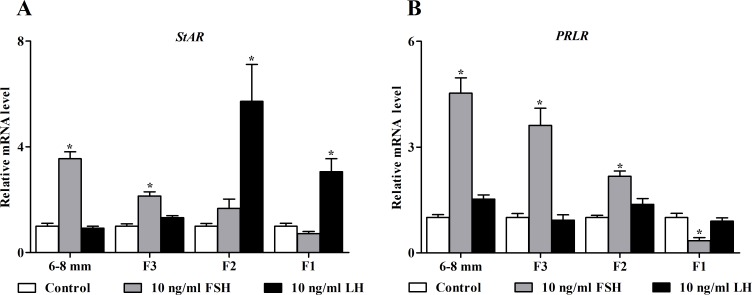
**Effects of gonadotropins on levels of *StAR* (A) and *PRLR* (B) transcripts in chicken granulosa cells of different size class follicles after a 24-h culture.** Relative expression level was normalized to *18S rRNA*. Data are expressed as fold differences ± SEM of six independent experiments using tissues from different hens and are compared to control cells of each follicular class. ^*^, *P* < 0.05 compared to control cells within each follicular class.

Since *PRLR* was minimally expressed in theca layers compared to granulosa layers in the hierarchical follicles (Fig 1B), we further investigated its regulation by gonadotropins in granulosa cells from the 6–8 mm (undifferentiated) follicles and F3-F1 (differentiated) follicles. We also evaluated the abundance of *StAR* transcript, which is regulated by gonadotropins, as a validation of our cell culture model. The effects of FSH and LH on expression of *StAR* and *PRLR* were different depending on the degree of granulosa cell differentiation. In agreement with data previously reported [3, 31], FSH stimulated a 3.5-fold increase (*P* < 0.05) in the mRNA levels of *StAR* in granulosa cells of the 6–8 mm follicles, whereas, this stimulatory effect was progressively reduced as the follicles matured to F1. As expected, LH did not alter *StAR* transcript levels (*P* > 0.05) in cells of either the 6–8 mm or F3 follicles, but increased its levels by 5.7- and 3.1-fold (*P* < 0.05) in F2 and F1 follicles, respectively (Fig 2A). In contrast, a similar pattern of FSH stimulation on *PRLR* mRNA expression was also observed in granulosa cells from the 6–8 mm to F1 follicles with the most inducible effect (a 4.5-fold increase, *P* < 0.05) in the 6–8 mm follicles, whereas, LH had no effect on *PRLR* transcription in cells from all follicular classes examined (*P* > 0.05; Fig 2B).

### Effects of PRL variants on basal and FSH-stimulated *PRLR* mRNA levels in chicken granulosa cells

**Fig 3:**
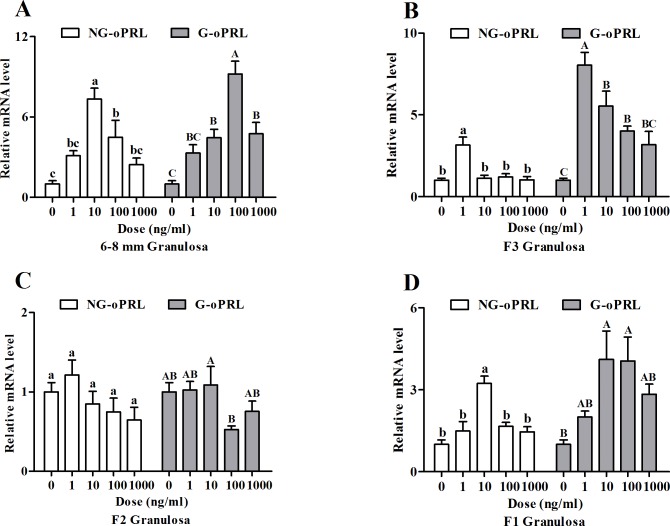
**Regulation of *PRLR* transcript by non-glycosylated (NG-) and glycosylated (G-) ovine PRL (oPRL) (0, 1, 10, 100 or 1000 ng/ml) in cultured granulosa cells of the 6–8 mm (A), F3 (B), F2 (C) and F1 (D) follicles after a 24-h culture.** Relative expression level was normalized to *18S rRNA*. Data are expressed as fold differences ± SEM of three independent experiments using tissues from different hens and are compared to control cells of each follicular class. Different letters indicate significant differences at *P* < 0.05.

In galliformes, levels of circulating PRL and the degree of glycosylation of PRL significantly vary during different reproductive stages. Hence, we investigated the effects of non-glycosylated (NG-) and glycosylated (G-) ovine PRL (oPRL) on *PRLR* expression in granulosa cells of follicles at different developmental stages. Granulosa cells of the 6–8 mm follicles were most sensitive to increasing concentrations of either isoform of PRL and a similar dose-response of *PRLR* mRNA levels was observed with maximal stimulatory effects of NG- and G-oPRL achieved at doses of 10 and 100 ng/ml (*P* < 0.05), respectively (Fig 3A). A similar pattern was observed in granulosa cells from F1 follicles (Fig 3D). In granulosa cells of F3 and F2 follicles, NG-oPRL had minimal effect on *PRLR* mRNA levels, whereas G-oPRL was stimulatory in F3 but tended to be inhibitory in F2 granulosa cells (Fig 3B and 3C).

**Fig 4:**
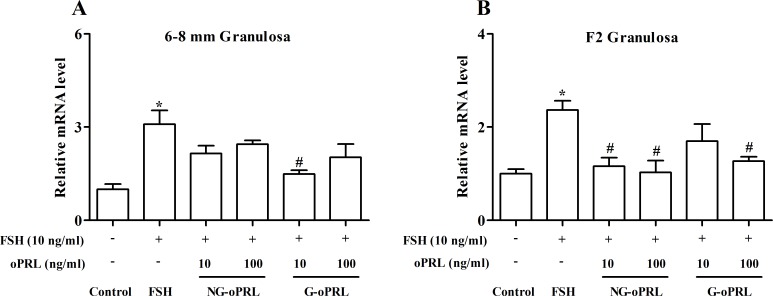
**Effects of non-glycosylated (NG-) and glycosylated (G-) ovine PRL (oPRL) (0, 10 or 100 ng/ml) on FSH-mediated *PRLR* mRNA expression in cultured granulosa cells of the 6–8 mm (A) and F2 (B) follicles after a 24-h culture.** Relative expression level was normalized to *18S rRNA*. Data are expressed as fold differences ± SEM of three independent experiments using tissues from different hens and are compared to control cells of each follicular class. ^*^, *P* < 0.05 compared to control cells; ^#^, *P* < 0.05 compared to cells only stimulated by 10 ng/ml FSH.

Since either FSH or PRL isoforms were involved in modulating levels of the *PRLR* transcript, we further examined the effects of their interaction in undifferentiated (6–8 mm) and differentiated (F2) granulosa cells. Both PRL isoforms inhibited FSH-induced expression of *PRLR* in granulosa cells of F2 follicles (*P* < 0.05), whereas, these effects were not significant except for 10 ng/ml G-oPRL in the 6–8 mm follicles (Fig 4).

### Role of PKA and PKC pathways in FSH-induced *PRLR* and *StAR* expression in undifferentiated granulosa cells

**Fig 5:**
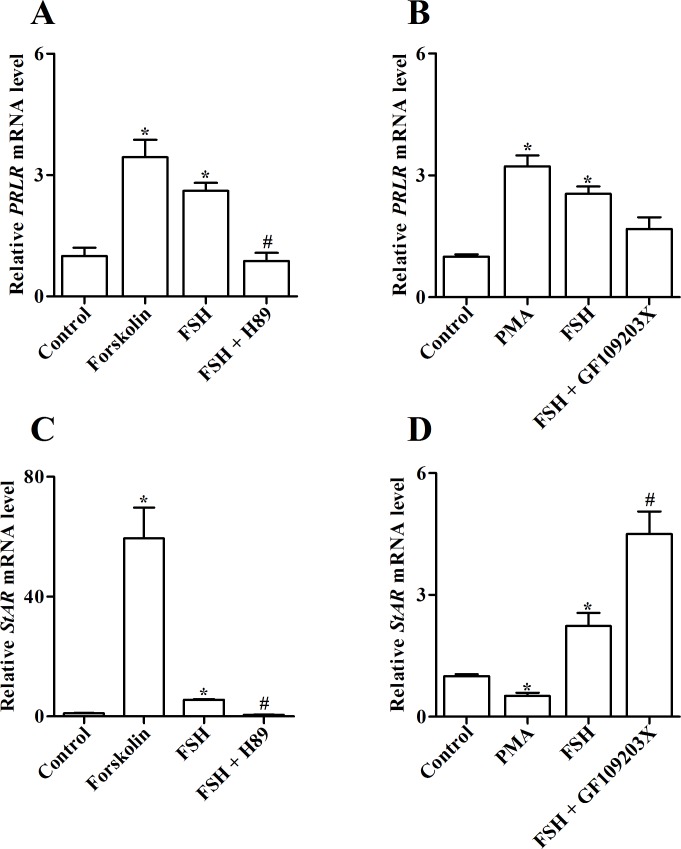
Role of PKA and PKC signaling pathways in FSH-induced *PRLR* and *StAR* mRNAs expression in cultured granulosa cells of prehierarchical (6–8 mm) follicles. (A and C) Changes in relative mRNA levels of *PRLR* (A) and *StAR* (C) after culture of granulosa cells for 4 h in the absence or presence of 10 ng/ml FSH in combination with PKA activator (10 μM Forskolin) or inhibitor (20 μM H89). (B and D) Changes in relative mRNA levels of *PRLR* (B) and *StAR* (D) in granulosa cells treated without or with 10 ng/ml FSH in combination with PKC activator (20 nM PMA) or inhibitor (10 μM GF109203X). Relative expression level was normalized to *18S rRNA*. Data are expressed as fold differences ± SEM of three independent experiments using tissues from different hens and are compared to control cells. ^*^, *P* < 0.05 compared to control cells; ^#^, *P* < 0.05 compared to cells only stimulated by 10 ng/ml FSH.

Since FSH is essential for the selection of follicles within the 6–8 mm cohort into the preovulatory hierarchy [5] and stimulated the greatest increase in *PRLR* and *StAR* transcript levels in granulosa cells of the 6–8 mm follicles, we sought to elucidate the cellular mechanisms underlying FSH-induced *PRLR* and *StAR* expression in such size class follicles. The role of PKA and PKC signaling pathways was firstly evaluated using pharmacological activators and inhibitors. The doses of PKA activator and inhibitor (*i*.*e*. Forskolin and H89, respectively) were derived from earlier studies in chickens [2, 32–34]. Consistent with previous results, FSH treatment increased *PRLR* and *StAR* mRNA levels in all cultured granulosa cells of the 6–8 mm follicles (*P* < 0.05; Fig 5). Furthermore, FSH-induced increase in levels of *PRLR* and *StAR* transcripts was mimicked by 10 μM Forskolin but was suppressed by 20 μM H89 (*P* < 0.05; Fig 5A and 5C).

Since different doses of PKC activator and inhibitor (*i*.*e*. PMA and GF109203X, respectively) have been used in chicken granulosa cells [31, 35, 36], the dose-response effect of either PMA or GF109203X on *PRLR* expression was first determined in the present study (S1 Fig). Compared to untreated cells, 20 nM PMA and 10 μM GF109203X effectively stimulated and inhibited *PRLR* mRNA levels (*P* < 0.05), respectively. These concentrations were used in subsequent experiments. Treatment with PMA enhanced basal levels of *PRLR* transcript similar to FSH while GF109203X had no effect on FSH-induced *PRLR* expression (Fig 5B). In contrast, PMA reduced basal *StAR* levels and the FSH-stimulated increase in *StAR* transcript was further enhanced by addition of GF109203X (*P* < 0.05; Fig 5D).

### Role of PI3K-Akt-mTOR and AMPK pathways in FSH-induced *PRLR* and *StAR* expression in undifferentiated granulosa cells

**Fig 6:**
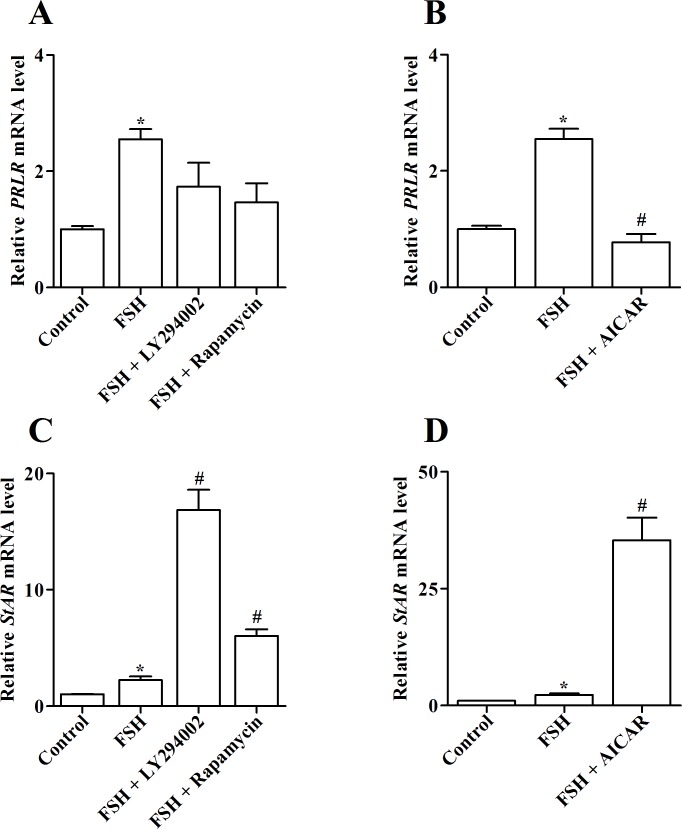
Role of PI3K-Akt-mTOR and AMPK signaling pathways in FSH-induced *PRLR* and *StAR* mRNAs expression in cultured granulosa cells of prehierarchical (6–8 mm) follicles. (A and C) Changes in relative mRNA levels of *PRLR* (A) and *StAR* (C) after culture of granulosa cells for 4 h in the absence or presence of 10 ng/ml FSH together with PI3K inhibitor (20 μM LY294002) or mTOR inhibitor (10 μM Rapamycin). (B and D) Relative *PRLR* (B) and *StAR* (D) mRNAs levels in granulosa cells cultured for 4 h in the absence or presence of 10 ng/ml FSH, or together with AMPK activator (1 mM AICAR). Relative expression level was normalized to *18S rRNA*. Data are expressed as fold differences ± SEM of three independent experiments using tissues from different hens and are compared to control cells. ^*^, *P* < 0.05 compared to control cells; ^#^, *P* < 0.05 compared to cells only stimulated by 10 ng/ml FSH.

Next, we determined the role of PI3K-Akt-mTOR and AMPK signaling pathways in FSH-induced increase in levels of *PRLR* and *StAR* transcripts. The doses of the PI3K inhibitor (LY294002) as well as the mTOR inhibitor (Rapamycin) were used according to recent studies in geese [37] and chickens [38], and the dose of the AMPK activator AICAR was based on a study in chicken granulosa cells [9]. Neither 20 μM LY294002 nor 10 μM Rapamycin altered FSH-induced *PRLR* expression (*P* > 0.05; Fig 6A), whereas it was suppressed by treatment with 1 mM AICAR (*P* < 0.05; Fig 6B). In contrast, either of LY294002 and Rapamycin further enhanced FSH-induced *StAR* transcript levels which were also raised by addition of AICAR (*P* < 0.05; Fig 6C and 6D).

### Abundance of ERK2 in chicken developing follicles and its role in FSH-induced expression of *PRLR* and *StAR* in undifferentiated granulosa cells

**Fig 7:**
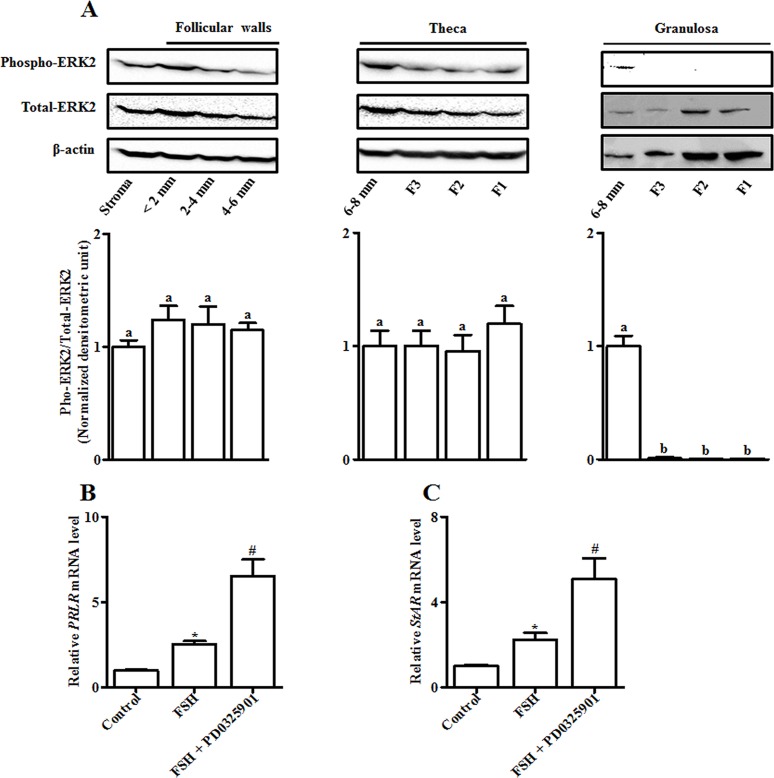
Abundance of ERK2 protein in chicken developing follicles and its role in FSH-induced *PRLR* and *StAR* expression in cultured granulosa cells of 6–8 mm follicles. (A) Top panel: Representative western blot analysis of phosphorylated and total ERK2 in the stroma and walls of prehierarchical (< 2, 2–4 and 4–6 mm) follicles as well as in theca and granulosa cell layers separated from the 6–8 mm and F3-F1 follicles. β-actin was used as loading control. Bottom panel: Quantitative analysis of relative protein abundance of phosphorylated ERK2 by densitometry using Image Lab software (Version 4.1, Bio-Rad laboratories). Relative protein abundance was normalized to total ERK2. Data are expressed as fold differences ± SEM compared to an appropriate tissue (n = 4 hens). Bars with different letters are significantly different at *P* < 0.05. (B and C) Changes in relative mRNAs levels of *PRLR* (B) and *StAR* (C) in granulosa cells treated without or with 10 ng/ml FSH, or together with the MEK inhibitor (1 μM PD0325901) after a 4-h culture. Relative mRNA expression level was normalized to *18S rRNA*. Data are expressed as fold differences ± SEM of three independent experiments using tissues from different hens and are compared to control cells. ^*^, *P* < 0.05 compared to control cells; ^#^, *P* < 0.05 compared to cells only stimulated by 10 ng/ml FSH.

Because ERK2 plays a crucial role in preventing premature differentiation in granulosa cells from chicken prehierarchical follicles [5], we then investigated its expression pattern in ovarian follicles during development as well as its role in FSH-induced *PRLR* and *StAR* expression in 6–8 mm granulosa cells. By using rabbit anti-mouse total- or phospho-ERK1/2 monoclonal antibodies, it was observed that only ERK2 protein with the size of ~ 42 kDa was detected. In the stroma and walls of < 6 mm follicles, levels of either total- or phospho-ERK2 remained unchanged (*P* > 0.05), except for a non-significant increase in ERK2 phosphorylation in < 4 mm follicles. Furthermore, the abundance of ERK2 in theca and granulosa cell layers from prehierarchical (6–8 mm) and preovulatory (F3-F1) follicles was also determined. ERK2 phosphorylation in theca layers did not alter as follicles matured (*P* > 0.05), but much higher levels of ERK phosphorylation were found in granulosa layers of the 6–8 mm follicles than those of F3-F1 follicles (*P* < 0.05; Fig 7A).

The dose of the MEK inhibitor PD0325901 was used according to its effective inhibition on ERK2 phosphorylation in granulosa cells of the 6–8 mm follicles (S2 Fig). Treatment with 1 μM PD0325901 further enhanced FSH-induced increase in levels of both *PRLR* and *StAR* transcripts (*P* < 0.05; Fig 7B and 7C).

### Effects of manipulation of several intracellular signaling pathways on basal and FSH-stimulated ERK2 phosphorylation in undifferentiated granulosa cells

**Fig 8:**
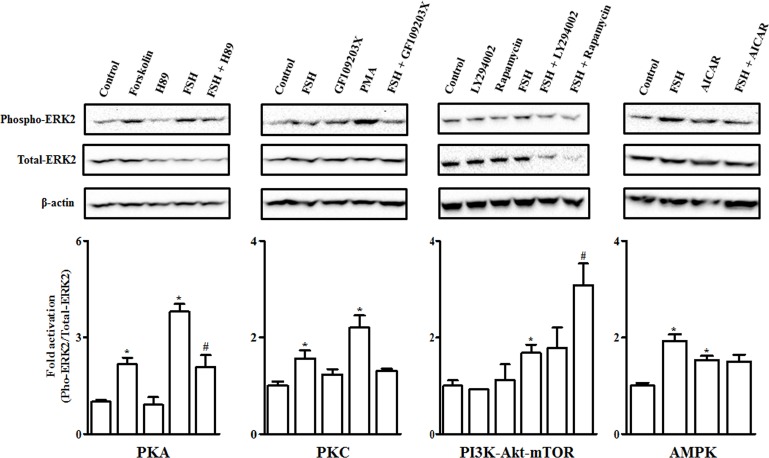
Effects of manipulation of several intracellular signaling pathways on basal and FSH-induced ERK2 phosphorylation in cultured granulosa cells of prehierarchical (6–8 mm) follicles. Top panel: Representative western blot analysis of phosphorylated and total ERK2 in granulosa cells treated without or with 10 ng/ml FSH in combination with PKA activator or inhibitor (10 μM Forskolin or 20 μM H89, respectively), or PKC activator or inhibitor (20 nM PMA or 10 μM GF109203X, respectively), or PI3K inhibitor (20 μM LY294002) or mTOR inhibitor (10 μM Rapamycin), or AMPK activator (1 mM AICAR). β-actin was used as loading control. Bottom panel: Quantitative analysis of relative protein abundance of phosphorylated ERK2 by densitometry using Image Lab software (Version 4.1, Bio-Rad laboratories). Relative protein abundance was normalized to total ERK2. Data are expressed as fold differences ± SEM of three independent experiments using tissues from different hens and are compared to control cells. ^*^, *P* < 0.05 compared to control cells; ^#^, *P* < 0.05 compared to cells only stimulated by 10 ng/ml FSH.

Since there is a difference in levels of ERK2 phosphorylation between undifferentiated and differentiated granulosa cells and crosstalk of ERK signaling with other signaling pathways commonly occurs in regulating ovarian steroidogenesis [39], we further investigated the effects of manipulation of other signaling pathways on basal and FSH-mediated ERK2 phosphorylation in granulosa cells of the 6–8 mm follicles. Compared to untreated cells, stimulation with 10 ng/ml FSH for 4 h stimulated phosphorylation of ERK2 (*P* < 0.05), which was mimicked by addition of 10 μM Forskolin. Inhibition of PKA by 20 μM H89 did not alter (*P* > 0.05) basal but suppressed (*P* < 0.05) FSH-induced ERK2 phosphorylation. In contrast, treatment with the PKC inhibitor GF109203X had no effects on both basal and FSH-stimulated ERK phosphorylation (*P* > 0.05). However, activation of PKC by 20 nM PMA increased basal levels of ERK phosphorylation (*P* < 0.05; Fig 8).

Furthermore, blockage of PI3K or mTOR signaling using 20 μM LY294402 or 10 μM Rapamycin, respectively, did not impair the activity of ERK2 when compared to control cells (*P* > 0.05). However, in the presence of FSH, Rapamycin further increased (*P* < 0.05) while LY294002 showed no effect on FSH-induced ERK2 activation (*P* > 0.05). Activation of AMPK signaling by 1mM AICAR stimulated basal (*P* < 0.05) but failed to further enhance FSH-induced ERK2 phosphorylation (*P* > 0.05; Fig 8).

## Discussion

In chickens, PRL has been shown to have direct effects on the ovary to modulate steroidogenesis yet the distribution of its receptor PRLR within the follicular hierarchy has not been quantified. In the current study we show that maximal expression of *PRLR* transcript was observed in the stroma and walls of follicles < 2 mm in diameter before a progressive decline with follicle maturation (Fig 1A). Since the smaller (< 2 mm) follicles represent the largest follicular class in the ovary and are thought to be the major source of estrogen [40] and exogenous PRL could suppress both basal and gonadotropin-stimulated estrogen production by cultured hen these small follicles [20, 41], it is likely that PRL may have a dominantly negative influence on steroidogenesis in the smaller follicles. However, such inhibitory effects by PRL may be attenuated or even become stimulatory dependent on its concentration and the stage of follicle development. Divergent effects of PRL upon steroidogenesis in porcine granulosa cells was demonstrated to be associated with the degree of cell differentiation [42]. Furthermore, in chickens, a stimulatory role for PRL in recruiting large white follicles into small yellow ones was suggested through immunizations against PRL or PRLR [19]. Dependent on the dose of PRL, the stage of follicle development and the stage of the ovulatory cycle, PRL could be either stimulatory or inhibitory on estradiol secretion by the theca layers or progesterone production by the granulosa layers of F3-F1 follicles [20]. Notably, in the preovulatory hierarchy, the *PRLR* transcript was more abundant in granulosa than theca layers (Fig 1B), implying a relatively more important role of PRL signaling in regulating progesterone production and progesterone-induced ovulation. Indeed, activity of 3β-hydroxysteroid dehydrogenase (3βHSD), a key enzyme in the progesterone biosynthetic pathway, was shown to be regulated by gonadotropins and PRL in granulosa cells of chicken F3-F1 follicles [43], and PRL tended to suppress LH-induced premature ovulation in chickens [44]. Accordingly, we speculated that varying levels of *PRLR* transcript during follicle development are related to changes in the process of follicular cell steroidogenesis as a result of their different responsiveness to gonadotropins and PRL.

Although there is a close relationship between gonadotropins and PRL in control of ovarian functions in many species of birds, to our knowledge, information about their actions in modulating PRL signaling through the PRLR at the cellular level remains scarce. The present study provides the first evidence for the independent and interactive effects of gonadotropins and PRL variants on expression of the *PRLR* gene by cultured chicken granulosa cells. These effects were different dependent on the stage of follicle development (*i*.*e*. the degree of granulosa cell differentiation). In undifferentiated granulosa cells, FSH induced the greatest increase in the mRNA levels of *PRLR* in parallel with that in the *StAR* transcript, whereas, this stimulatory effect gradually declined in F3 and F2 granulosa cells and eventually became inhibitory in F1 cells. In contrast, LH had no effect on *PRLR* mRNA expression in granulosa cells from all size class follicles examined although it did increase levels of *StAR* transcript in F2 and F1 granulosa cells (Fig 2). The magnitude of gonadotropin (FSH or LH) stimulation on expression of *StAR* unequivocally supported the observation that there is a shift from FSHR dominance in undifferentiated follicles to LHR dominance in the preovulatory hierarchy [5]. In addition to the *StAR* gene, elevated levels of P450scc and LHR as well as progesterone production induced by FSH are also recognized as markers of granulosa cell differentiation [5]. Although the role for PRL signaling in modulating granulosa cell differentiation in galliformes is not known, the greatest stimulatory effect of FSH on *PRLR* transcription in chicken undifferentiated granulosa cells may suggest a positive effect of PRL by potentiating the actions of FSH during follicle selection. These novel data are supported by the observations in rat granulosa cells where FSH promoted the expression and formation of functional PRLRs *in vivo* and *in vitro* and PRL enhanced FSH-induced progesterone production by amplifying the expression of StAR, P450scc and 3βHSD [45, 46].

In galliformes, the ratio of circulating G- to NG-PRL significantly varies during different stages of the reproduction cycle [27, 28]. Stimulation of undifferentiated granulosa cells with either variant produced a similar effect in inducing upregulation of *PRLR* transcript, whereas, G-PRL had a dominant effect in the hierarchal follicles (Fig 3). In galliformes that express incubation behaviour, hyperprolactinemia is associated with large shift in absolute and relative levels of G-PRL (from about 30% during egg laying to about 70% during incubation behaviour). Hens typically lay 3–5 eggs from the time the behaviour is first expressed until termination of lay and involution of the ovary. Thus, it would appear that hens oviposit the dominant follicles without recruiting replacements during this transitional phase. It is possible that these increasing levels of G-PRL induce increased PRLR and hence sensitivity to the inhibitory effects of PRL. In many species, G-PRL is reported to have decreased binding activity and consequently lower biological activity. However, avian receptors have a duplicated extracellular domain [26, 47]. NG- and G-PRL may interact differently within these ligand binding domains to affect signal transduction.

Furthermore, FSH alone up-regulated the expression of *PRLR* but this effect was reduced by the inclusion of either PRL isoform especially in the F2 granulosa cells (Fig 4). These data indicated an antagonistic effect of PRL on the actions of FSH in regulating *PRLR* expression in chicken granulosa cells and the magnitude of such inhibition is probably dependent on the concentration of PRL and the degree of granulosa cell differentiation. A role for PRL in regulating the actions of FSH was previously reported in porcine granulosa cells where lower levels of PRL enhanced FSH-induced FSHR binding but higher levels of PRL led to a decrease in the number of FSHR [48]. Furthermore, locally secreted growth factors such as the BMP system was involved in modifying the actions of PRL in FSH-induced steroid production in rat granulosa cells [45]. Thus, the inhibitory effect of PRL on FSH-induced *PRLR* expression in chicken granulosa cells may be related to its interaction with FSH signaling via modifying the FSHR binding as well as a potential negative feedback by other unknown autocrine/paracrine growth factors such as the BMP system. Taken together, these results indicated that expression of *PRLR* in granulosa cells is cooperatively regulated by gonadotropins and circulating PRLs in dose- and follicular size-dependent manners. However, the relationship between FSH and PRL signaling on regulation of PRLR expression during follicle development remains unclear, hence any potential networking between these pathways warrants further investigation.

Thereafter, we further investigated the role of several signaling pathways in FSH-induced PRLR and StAR expression in chicken undifferentiated granulosa cells. As in mammals, the conserved binding sites for several transcription factors such as CCAAT/enhancer-binding protein (C/EBP) and SP1 are also characterized in the promoter region of chicken *PRLR* [25]. FSH is able to modulate the expression and activity of both C/EBP and SP1 via multiple signaling pathways in a variety of cell types [49, 50], thereby regulating the transcription of *PRLR*. Use of activators to activate the PKA or PKC pathways both increased basal levels of *PRLR* transcript, whereas, only the inhibition of PKA abolished FSH-induced *PRLR* expression (Fig 5A and 5B), indicating that FSH regulates PRLR expression in a PKA-dependent manner. Activation of PKA increased *StAR* transcript levels while its inhibition blocked FSH-induced *StAR* expression. In contrast, addition of the PKC activator (PMA) reduced *StAR* levels but inhibition of PKC by GF109203X augmented the stimulatory effects of FSH on *StAR* expression (Fig 5C and 5D). These results confirm previous findings showing that PKA is positively involved in but PKC is negatively involved in granulosa cell differentiation in chickens [31, 51]. In addition, neither LY294002 nor Rapamycin suppressed FSH-induced *PRLR* levels but both did potentiate the stimulation of FSH on *StAR* expression (Fig 6A and 6C). Since both pro-survival and proliferative effects of the PI3K-Akt-mTOR signaling have been demonstrated in chicken or goose granulosa cells [37, 52, 53], it is hypothesized that in hen undifferentiated follicles, suppression of this pathway may depress proliferation and promote steroidogenesis to initiate granulosa cell differentiation. As a cellular energy sensor, AMPK is widely expressed in chicken preovulatory granulosa cells and is involved in FSH- or IGF-regulated steroidogenesis via modulation of several steroidogenic enzymes expression such as StAR and 3βHSD [9, 54]. It was observed that activation of AMPK by AICAR reduced FSH-induced *PRLR* mRNA levels but enhanced FSH-induced *StAR* expression in chicken undifferentiated granulosa cells (Fig 6B and 6D), suggesting that AMPK also participates in chicken granulosa cell differentiation through regulation of PRLR and StAR.

The actions of these signaling pathways in FSH-induced PRLR and StAR expression may be implicated in the regulation of ERK2 phosphorylation. As reported by other researchers [3, 38], only ERK2 was present in chicken ovarian tissues examined (Fig 7A). The widespread expression of ERK2 in walls of < 6 mm follicles as well as in theca and granulosa cell layers from > 6 mm follicles imply that it plays a universal role in the hierarchy. However, greater levels of ERK2 phosphorylation were detected in undifferentiated (6–8 mm) than differentiated (F3-F1) granulosa cells, suggesting different actions of ERK2 between prehierarchical and preovulatory follicles. Indeed, ERK2 precludes premature differentiation in granulosa cells from prehierarchical follicles through inhibition of steroidogenesis, whereas, in preovulatory follicles ERK2 enhanced LH-induced progesterone production [3, 31, 35]. In agreement with the effects of the two MEK inhibitors (*i*.*e*. U0126 and PD98059) on FSH-induced StAR levels in chicken granulosa cells [3, 51], inhibition of ERK2 by PD0325901 enhanced FSH-stimulated *PRLR* and *StAR* expression in undifferentiated granulosa cells (Fig 7B and 7C), further confirming a predominantly negative role of ERK2 in follicles prior to selection. Similar to the observations in mammalian immature granulosa cells [55], in chicken undifferentiated granulosa cells FSH treatment induced an increase in ERK2 phosphorylation which was abolished by addition of PD0325901 (S1 Fig). Although FSH-induced ERK2 phosphorylation appeared paradoxical with the inhibitory effect of ERK2 on FSH-stimulated steroidogenesis during the prehierarchical phase, there is evidence that transient activation and subsequent termination of ERK2 is required for the differentiation of granulosa cells in chickens [56]. In chicken undifferentiated granulosa cells, stimulation of TGFβ1 on *FSHR* transcription is abrogated via activation of ERK2 by the EGF receptor ligands (*e*.*g*. TGFα, betacellulin) [5], whereas, temporal activation of ERK2 is required for TGFα or betacellulin-induced *TGFβ1* expression which implicates ERK2-mediated expression of MAPK phosphatases [56, 57]. Likewise, a feedback mechanism whereby ERK2 is initially activated and subsequently terminated by FSH via modulation of phosphatase activity may exist in chicken prehierarchical follicles, thereby contributing to granulosa cell differentiation by controlling the expression of several differentiation-inducing genes such as *FSHR*, *TGFβ1* and *PRLR*.

In accordance with the effects of 8-bromo-cAMP [55], Forskolin increased basal levels of ERK2 phosphorylation but H89 reduced FSH-induced ERK2 activation (Fig 8), indicating that in chicken undifferentiated granulosa cells FSH stimulates ERK2 activation in a cAMP/PKA-dependent manner. Activation of PKC by PMA increased basal levels of ERK2 phosphorylation but inhibition of PKC by GF109203X did not alter FSH-induced ERK2 phosphorylation, which corresponded with their effects on PRLR expression, suggesting that PMA mediates *PRLR* transcription through the PKC/ERK2 pathway. Furthermore, the PI3K inhibitor, LY294002, had no effect but the mTOR inhibitor, Rapamycin, enhanced FSH-induced ERK phosphorylation (Fig 8). In chicken myoblasts 50 μM LY294002 reduced basal and insulin-induced ERK phosphorylation [38]. This discrepancy may be due to differences in the cell type, the dose of LY294002 and the hormone examined. Our results indicate that blockade of the mTOR signaling may to some extent promote granulosa cell differentiation by inhibiting proliferation and inducing differentiation via transient activation of ERK. Addition of the AMPK activator (AICAR) increased basal levels of ERK phosphorylation and resulted in a nonsignificant decrease in FSH-induced ERK phosphorylation in undifferentiated granulosa cells (Fig 8). The stimulatory effect of AICAR on basal ERK phosphorylation as well as its divergent effects on FSH-induced ERK phosphorylation have been reported in hen F1 and F3 plus F4 follicles [9]. These observations suggest that differential effects of the AMPK signaling on FSH-induced steroidogenesis in granulosa cells are dependent on the stage of follicle maturation and are involved in regulation of ERK activity.

In conclusion, our data suggest that *PRLR* was differentially expressed and regulated by FSH or PRL variants independently, or in combination in the follicular hierarchy. In undifferentiated granulosa cells, FSH-induced *PRLR* and *StAR* expression was mediated by multiple signaling pathways including PKA, PKC, PI3K, mTOR and AMPK. All of these pathways may also converge to modulate ERK activity to regulate granulosa cell differentiation.

## Supporting Information

S1 Fig Dose optimization of the activator (PMA) and inhibitor (GF109203X) of PKC in regulation of *PRLR* expression in cultured granulosa cells of prehierarchical (6–8 mm) follicles. (A and B) Changes in relative *PRLR* mRNA levels in granulosa cells responding to different doses of either PMA (0, 5, 10, 20, 40 or 80 nM) (A) or GF109203X (0, 1.25, 2.5, 5, 10 or 20 μM) (B) after a 4-h culture. Relative expression level was normalized to *18S rRNA*. Data are expressed as mean ± SEM of three independent experiments using tissues from different hens and are compared to control cells. ^*^, *P* < 0.05; compared to control cells.
(TIF)

S2 Fig Representative western blot analysis of phosphorylated and total ERK2 in cultured granulosa cells of prehierarchical (6–8 mm) follicles treated without or with 10 ng/ml FSH, or in combination with the MEK inhibitor (1 μM PD0325901). β-actin was used as loading control.
(TIF)
