# Supplementary figures and images for: Regulatory Mechanisms Underlying the Expression of Prolactin Receptor in Chicken Granulosa Cells
Source: PLoS One. 2017 Jan 20;12(1):e0170409. doi: 10.1371/journal.pone.0170409 (PMC5249103; doi:10.1371/journal.pone.0170409)

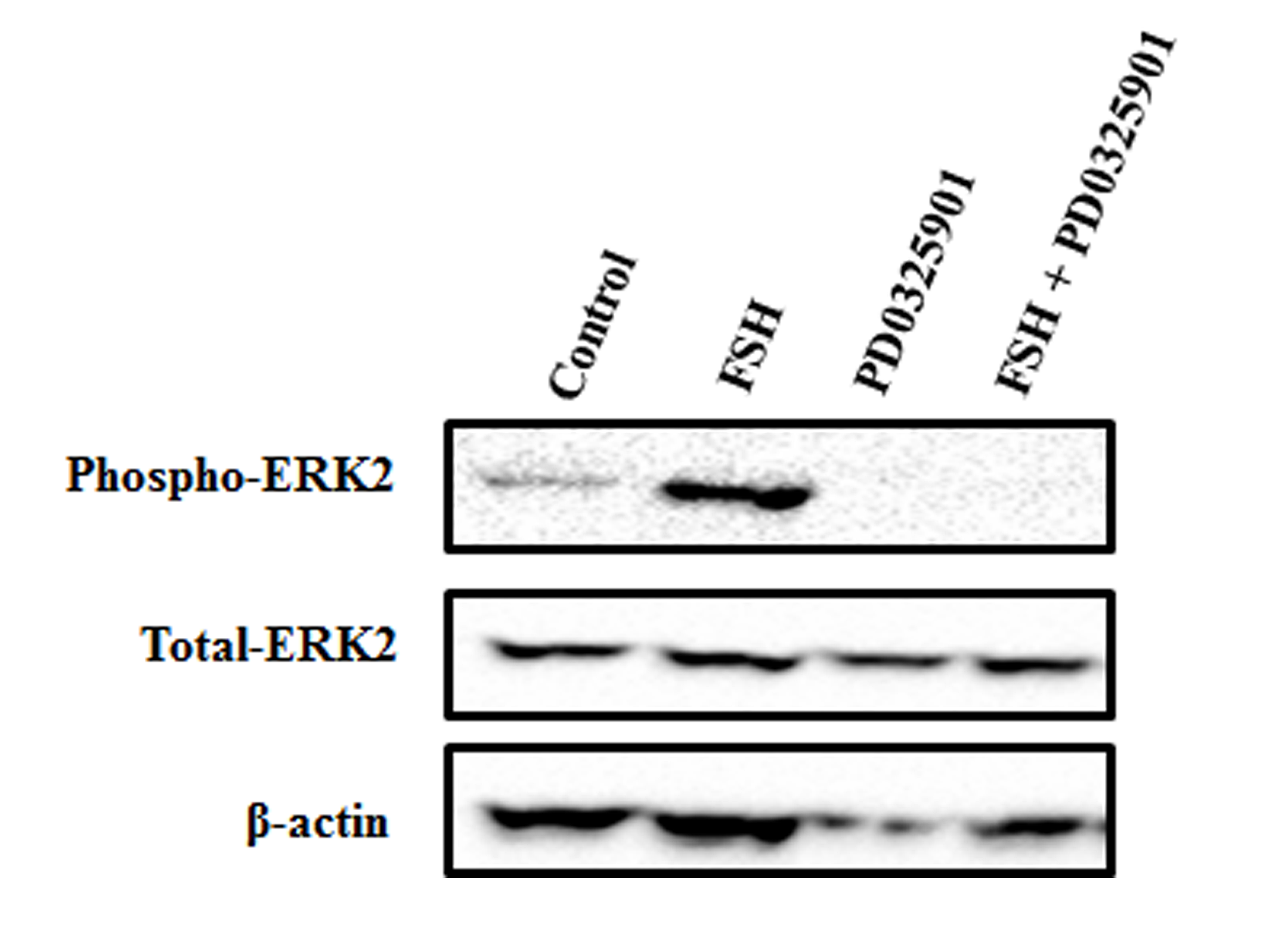

Supplement: S2 Fig — β-actin was used as loading control. (TIF) [file pone.0170409.s002.tif]
